# Short-term prognostic models for severe acute kidney injury patients receiving prolonged intermittent renal replacement therapy based on machine learning

**DOI:** 10.1186/s12911-023-02231-2

**Published:** 2023-07-24

**Authors:** Wenqian Wei, Zhefei Cai, Lei Chen, Weijie Yuan, Yingle Fan, Shu Rong

**Affiliations:** 1 Department of Nephrology, Shanghai General Hospital, Shanghai Jiao Tong University School of Medicine, Shanghai, China; 2 Hangzhou Dianzi University, Hangzhou, China

**Keywords:** Machine learning, Prolonged intermittent renal replacement therapy, Severe acute kidney injury, Prognosis prediction models, Correlation measurement

## Abstract

**Background:**

As an effective measurement for severe acute kidney injury (AKI), the prolonged intermittent renal replacement therapy (PIRRT) received attention. Also, machine learning has advanced and been applied to medicine. This study aimed to establish short-term prognosis prediction models for severe AKI patients who received PIRRT by machine learning.

**Methods:**

The hospitalized AKI patients who received PIRRT were assigned to this retrospective case-control study. They were grouped based on survival situation and renal recovery status. To screen the correlation, Pearson’s correlation coefficient, partial ETA square, and chi-square test were applied, eight machine learning models were used for training.

**Results:**

Among 493 subjects, the mortality rate was 51.93% and the kidney recovery rate was 30.43% at 30 days post-discharge, respectively. The indices related to survival were Sodium, Total protein, Lactate dehydrogenase (LDH), Phosphorus, Thrombin time, Liver cirrhosis, chronic kidney disease stage, number of vital organ injuries, and AKI stage, while Sodium, Total protein, LDH, Phosphorus, Thrombin time, Diabetes, peripherally inserted central catheter and AKI stage were selected to predict the 30-day renal recovery. Naive Bayes has a good performance in the prediction model for survival, Random Forest has a good performance in 30-day renal recovery prediction model, while for 90-day renal recovery prediction model, it’s K-Nearest Neighbor.

**Conclusions:**

Machine learning can not only screen out indicators influencing prognosis of AKI patients receiving PIRRT, but also establish prediction models to optimize the risk assessment of these people. Moreover, attention should be paid to serum electrolytes to improve prognosis.

**Supplementary Information:**

The online version contains supplementary material available at 10.1186/s12911-023-02231-2.

## What’s already known about this topic?

- PIRRT is gradually applied in AKI patients due to its combination of different hemodialysis modes.
- As a result of artificial intelligence advancement, machine learning has been applied in AKI patients to establish a warning model.

## Research gap

- Studies establishing prognosis prediction models of AKI patients receiving PIRRT by machine learning are scarce. Our research filled a research gap of prognosis prediction models for these patients.

## What does this article add?

- Sodium, Total protein, Lactate dehydrogenase (LDH), Phosphorus, Thrombin time, Liver cirrhosis, chronic kidney disease stage, number of vital organ injuries, and AKI stage were associated with mortality, while Sodium, Total protein, LDH, Phosphorus, Thrombin time, Diabetes, peripherally inserted central catheter and AKI stage were selected to predict the 30-day renal recovery.
- Naive Bayes has a good performance in the prediction model for survival, Random Forest has a good performance in 30-day renal recovery prediction model, while for 90-day renal recovery prediction model, it’s K-Nearest Neighbor.

## Introduction

Acute kidney injury (AKI) is one of the most common clinical issues worldwide. AKI occurs in approximately 10–15% of patients admitted to hospital, while its incidence in intensive care has been reported in more than 50% of patients [1, 2].Severe AKI is a common fatal disease that seriously affects patients’ prognosis. Several multinational and multicenter international cross-sectional studies have confirmed that the increase in AKI severity is related to hospital mortality [3, 4]. Renal replacement therapy (RRT) is an essential and highly efficient action for severe AKI. As a hybrid RRT mode, prolonged intermittent renal replacement therapy (PIRRT) has been gradually applied in clinical practice because of its combination of continuous renal replacement therapy (CRRT) and intermittent hemodialysis (IHD) [5, 6]. However, only a few studies have evaluated the prognosis prediction model of patients receiving PIRRT.

The advancement of machine learning has led to the emergence of risk prediction models based on artificial intelligence in medicine area. Some studies have attempted to establish a multidisciplinary and cross-ward unified early warning model for AKI [7, 8]. However, the use of machine learning to establish prognosis prediction models for PIRRT is rare.

Thus, the research aimed to concentrated on the indicators before PIRRT onset, build short-term prognosis prediction models for severe AKI patients receiving PIRRT by machine learning, provide new ideas for risk assessment and ensure timely treatment of high-risk patients, which is of great significance to improve prognosis.

## Methods

### Population

AKI patients who received PIRRT from January 2012 to October 2018 at the Shanghai General Hospital, Shanghai Jiao Tong University School of Medicine, Shanghai, China, were included. They were grouped based on 30-day survival situation. The surviving patients were divided into kidney recovery and non-recovery groups at 30- and 90-day post-discharge, respectively. The recovery of renal function was defined as decreased Scr combined with discontinuation of any form of RRT or urine volume > 800 ml/24 h [9]. The follow-up was carried out by phone and outpatient visits. The Ethics Committee of Shanghai General Hospital, Shanghai Jiao Tong University School of Medicine, approved the study. The need for informed individual consent was waived by this committee.

### Inclusion criteria

① AKI patients (according to the 2012 Kidney Disease: Improving Global Outcomes (KDIGO) definition of AKI [1]); ② PIRRT was treated. We implemented common PIRRT scheme [10] with a Prismaflex M60 CRRT system and a 0.6 m^2^ AN69 dialysis membrane of Baxter, Deerfield, IL, USA. Patients underwent PIRRT 3–7 times a week with a duration of 4–6 h per time.

### Exclusion criteria

① Chronic kidney disease (CKD) stage 4/5 patients or maintenance dialysis patients; ② Other RRT modes; ③ Out of contact; ④ Data missing, including cases of low data quality and significant data(creatinine, urine volume) loss. For cases of low data quality, we defined it as cases with the absence of ≥ 20% of all research indicators, and patients who died within 48 h after the first PIRRT.

### Observed variables

As we reported before, the indicators we focused on include [10]: ① Basic information: gender, age, AKI etiology and stage; ② History of important chronic diseases: Hypertension, Diabetes, Coronary heart disease(CHD), CKD, Chronic obstructive pulmonary disease (COPD) and so on; ③ Laboratory tests(before first PIRRT): routine test of blood, liver function, renal function(including serum creatinine, estimated Glomerular filtration rate), serum electrolytes, coagulation indicators and so on; ④ Medication : diuretics, antihypertensive agents, lipid-lowering drugs, etc.; ⑤Other factors: including peripherally inserted central catheter (PICC), the number of vital organ injuries, etc.; ⑥PIRRT related parameters: PIRRT times, PIRRT frequency; ⑦ Follow-up indices: serum creatinine (Scr) and urine volume.

### Statistical analysis and data processing

#### Baseline analysis

Quantitative variables were described by mean ± standard deviation or median and quartile, while a t-test or non-parametric test was used according to the distribution of data. Qualitative variables were described by the number of cases and percentages of each category, while the chi-square and Fisher’s tests were used according to the applicable conditions of the test. P < 0.05 indicated a statistically significant difference. The data were analyzed using SPSS 25 (IBM, Armonk, NY, USA) and python 3.7.4.

#### Prognostic analysis

Firstly, research indicators were divided into two categories: categorical data and measurement data. For the measurement data, the partial ETA square was selected for correlation measurement; for categorical variables, the chi-square test was selected. Then, the indices related to survival outcome and 30-day and 90-day renal recovery were selected, respectively. The value of the partial ETA test > 0.16 indicated a strong correlation [11]. A P-value < 0.05 indicated a correlation between variables for the chi-square test.

Secondly, the correlation among selected indices was evaluated. For measurement data-measurement data, Pearson’s correlation coefficient was selected; the absolute value 0.8–1.0 indicated a very strong correlation, 0.6–0.8 indicated a strong correlation, and 0.4–0.6 was a medium correlation [12]. For the categorical data-categorical data, the chi-square test was selected. For the measurement data-categorical data, the partial ETA square was applied.

Finally, through correlation screening, the obtained uncorrelated indicators and prognostic outcomes were input into machine learning models, including Logistic Regression, Support Vector Machines (SVM), K-Nearest Neighbor (KNN), Naive Bayes, Perceptron, Stochastic Gradient Descent (SGD), Decision Tree, Perceptron and Random Forest for training, and the prognosis prediction models of patients with severe AKI who received PIRRT were obtained.

**Fig. 1:**
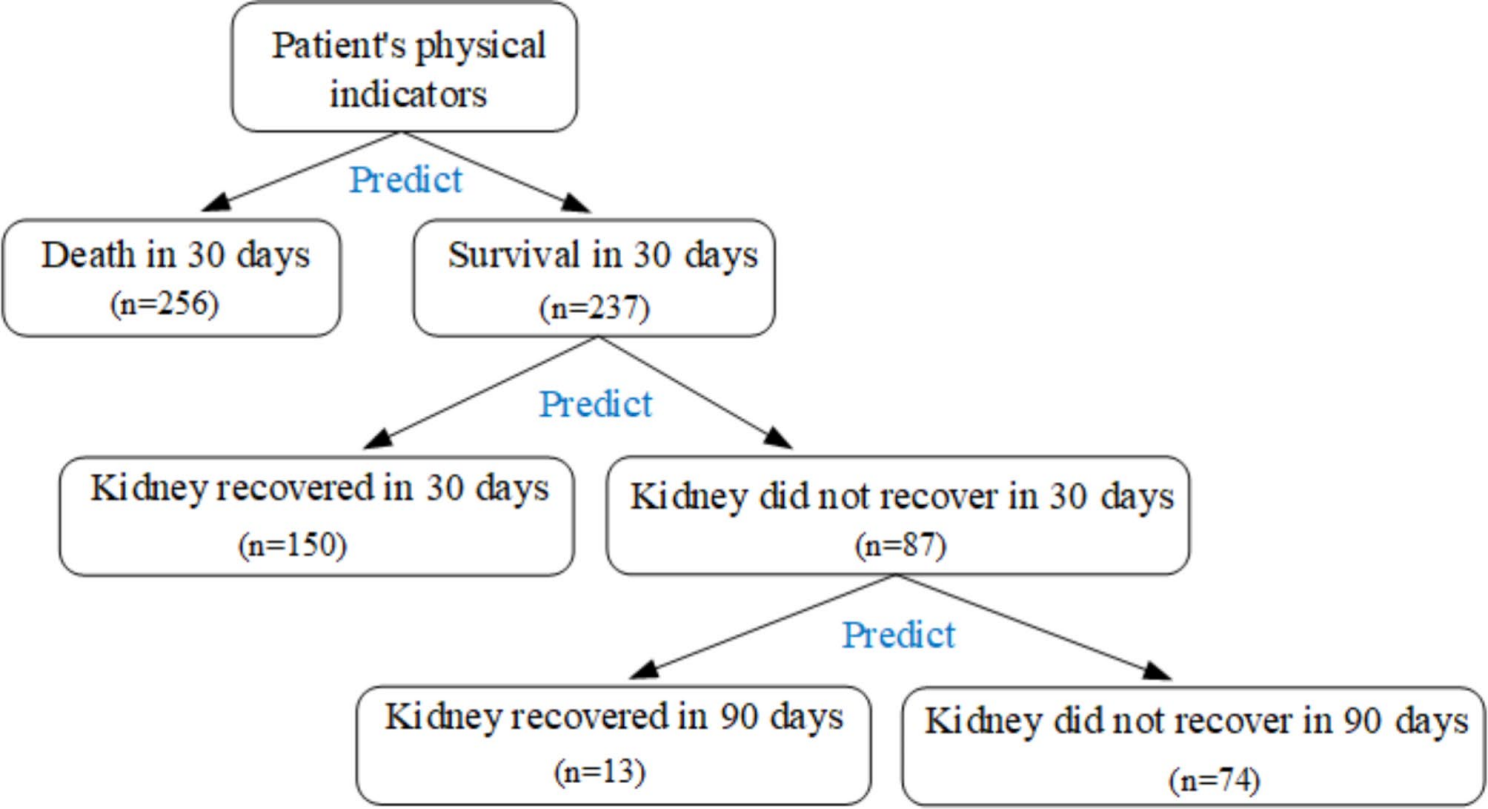
Flowchart of the construction of the prognosis prediction model

This study predicted the prognosis of patients according to the sequence of survival outcomes and 30- and 90-day renal recovery (Fig. 1).

## Results

### Baseline information of the subjects

A total of 493 subjects, including 340 (68.97%) males and 153(31.03%) females, had an average age of 61.6 ± 16.6 years. Eventually, 256 (51.93%) patients died, and 237 (48.07%) patients survived during the follow-up. Among surviving patients, 150 (30.43%) patients recovered at 30 days and 163(33.06%) at 90 days, respectively. The basic information of the subjects were shown in Table 1.

**Table 1:** Baseline information of subjects

	30-day death group (n = 256)	30-day survival group (n = 237)	P
Male, n (%)	175 (68.4)	165 (69.6)	0.762
Age, year, mean ± SD	65.6 ± 14.9	57.2 ± 17.2	<0.001
Hypertension (%)	101(57.7)	75 (31.64)	0.071
Diabetes (%)	62 (35.4)	44(18.6)	0.127
CHD (%)	28 (10.9)	27(11.4)	0.873
COPD (%)	8 (3.1)	6 (2.5)	0.692
Malignancy (%)	54 (21.09)	43 (18.1)	0.410
Liver cirrhosis (%)	26(10.2)	10 (4.2)	0.011
CKD			0.090
no (%)	222 (86.7)	187 (78.9)	
1 (%)	7 (2.7)	14 (5.9)	
2 (%)	2 (0.8)	10 (4.2)	
3 (%)	25(9.8)	26 (11.0)	
Potassium	4.60 ± 2.10	4.35 ± 1.14	0.108
Chlorine	104.19 ± 8.63	102.28 ± 6.83	0.007
Sodium	141.51 ± 8.71	138.21 ± 6.91	<0.001
Calcium	1.91 ± 0.35	2.00 ± 0.32	0.002
Magnesium	0.94 ± 0.27	0.94 ± 0.28	0.958
Phosphorus	1.88 ± 1.10	1.72 ± 0.87	0.086
Serum creatinine	394.92 ± 209.90	415.60 ± 275.53	0.347
GFR	12.56(8.30,22.90)	14.00(7.50,31.33)	0.452
Uric acid	510.33 ± 239.22	455.94 ± 211.98	0.008
Urea	27.48 ± 18.20	22.66 ± 14.73	0.001
Prothrombin time	19.24 ± 12.26	16.41 ± 10.80	0.007
Thrombin time	23.55 ± 14.70	19.99 ± 7.65	0.001
Total protein	54.81 ± 10.71	58.82 ± 10.45	<0.001
LDH	996(542,2150)	687(432,1560)	<0.001

### Experimental process

**Fig. 2:**
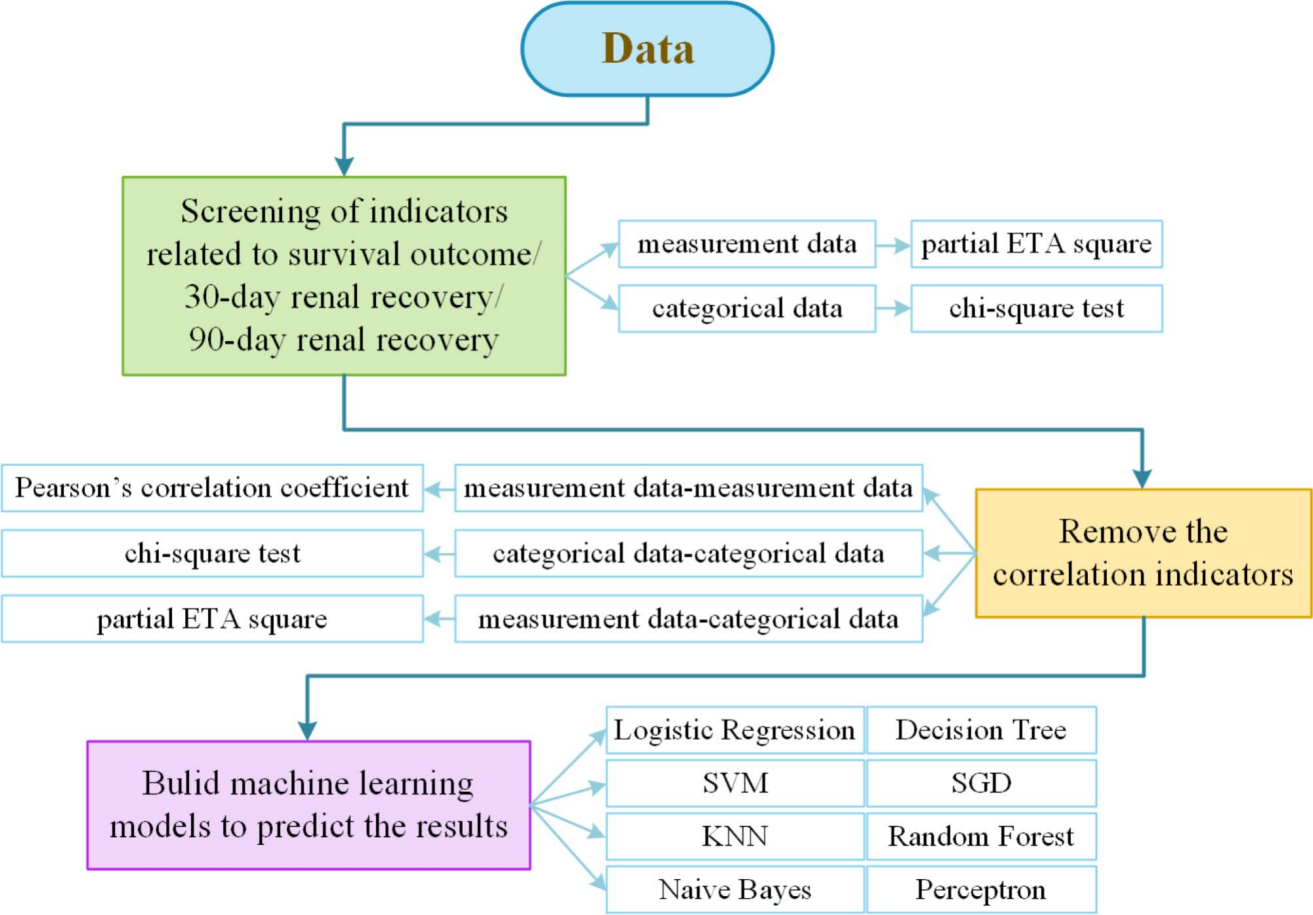
Logistic regression flowchart

The short-term prognosis prediction models for severe AKI patients receiving PIRRT were built according to the steps shown in Fig. 2. The algorithm in steps format was shown in Table 2.

**Table 2:** Algorithm: Framework of the survival prediction process

Algorithm: Framework of the survival prediction process
Using partial ETA square or chi-square test to screen the indicators related to survival;
**If** indicators do relate with survival outcomes:
Using Pearson’s correlation coefficient or chi-square test to measure the pairwise correlation of indicators;
**If** indicators are not related to each other in their respective categories:
Analyze the correlation between those measurement indices and categorical variables;
Select the final indices which can predict the survival;
**end**
**end**
Using machine learning methods, such as Logistic Regression, Support Vector Machines, K-Nearest Neighbor, Naive Bayes, Perceptron, Stochastic Gradient Descent, Decision Tree and Random Forest to predict the survival outcomes.

#### Screening of correlation indicators

1. Screening of indicators related to survival outcomes.

A correlation between patient survival outcome and the above observation indices was established using a partial ETA square for measurement data and a chi-square test for categorical variables. The results were shown in Table 3. Indices not included in the table were not related to survival outcomes.

**Table 3:** Correlation between indices and survival outcome

	Urea	Creatinine	GFR	Platelet hematocrit
Survival	0.888	0.958	0.913	0.331
	Hemoglobin	Sodium	Chlorine	Hematocrit
Survival	0.420	0.374	0.373	0.349
	Total protein	Potassium	Globulin	LDH
Survival	0.630	0.405	0.454	0.757
	Uric acid	Albumin	Calcium	Magnesium
Survival	0.856	0.455	0.263	0.219
	Phosphorus	Prothrombin time	Thrombin time	
Survival	0.449	0.312	0.324	
	ward type	cirrhosis	CKD stage	Hypertension
Survival	< 0.001	0.011	0.019	0.002
	diuretic	CCB	Albumin supplement	PIRRT frequency
Survival	0.021	0.002	< 0.001	0.001
	nephropathy history	infection	number of important organ injuries	AKI stage
Survival	0.021	0.004	< 0.001	< 0.001

Pearson’s correlation coefficient/chi-square test was used to measure the pairwise correlation between measurement data-measurement data and categorical variables-categorical variables retrospectively, and five measurement indices and the four categorical variables were selected. Subsequently, the correlation between those five measurement indices and four categorical variables was analyzed again (Table 4). After screening, all 9 indices were used to predict the survival outcomes: Sodium, Total protein, LDH, Phosphorus, Thrombin time, liver cirrhosis, CKD stage, number of vital organ injuries, and AKI stage.

**Table 4:** Correlation between measurement indicators and categorical variables (partial ETA square)

	Cirrhosis	CKD stage	Number of organ injuries	AKI stage
Sodium	0.002	0.016	0.013	0.002
Total protein	0.023	0.007	0.005	0.028
LDH	0.003	0.009	0.017	0.008
Phosphorus	0.008	0.013	0.039	0.040
Thrombin time	0.004	0.005	0.028	0.001

(2) Screening of indicators related to 30-day renal recovery.

The same method was used to screen out the indicators related to 30-day renal recovery. The correlation between indices and 30-day renal recovery was shown in Additional file 1. After measuring the pairwise correlation between measurement data-measurement data and categorical variables-categorical variables, 5 measurement indices and 3 categorical variables were identified. Eventually, all these 8 indices, including Sodium, Total protein, LDH, Phosphorus, Thrombin time, Diabetes, PICC, and AKI stage were selected to predict the 30-day renal recovery (Table 5).

**Table 5:** Correlation between measurement and categorical indicators (partial ETA square)

	Diabetes	PICC	AKI stage
Sodium	< 0.001	< 0.001	0.002
Total protein	0.008	0.011	0.028
LDH	0.007	0.004	0.008
Phosphorus	< 0.001	0.003	0.040
Thrombin time	< 0.001	0.001	0.001

(3) Screening of indicators related to 90-day renal survival outcome.

For 90-day renal function condition, the same analysis was utilized. The correlation between indices and 90-day renal recovery was shown in Additional file 1. After measuring the pairwise correlation between data, six measurement indices and two categorical variables were identified. All eight indices, including Hematocrit, Chlorine, Total protein, Uric acid, blood Phosphorus, Prothrombin time, CKD stage and diabetes, were selected (Table 6).

**Table 6:** Correlation between measurement and categorical indicators (partial ETA square)

	CKD stage	Diabetes
Hematocrit	0.016	0.001
Chlorine	0.008	< 0.001
Total protein	0.007	0.008
Uric acid	0.013	0.032
Phosphorus	0.013	< 0.001
Prothrombin time	0.017	0.001

### Establishment and verification of the prognosis prediction models

Based on the above-selected indicators, different machine learnings, including Logistic Regression, SVM, KNN, Naive Bayes, Perceptron, SGD, Decision Tree, Perceptron and Random Forest were used to build the prediction models.

In order to verify the model performance, we used the Accuracy (ACC), Precision, Recall, F1 and Area Under Curve (AUC) indicators. These indicators can be calculated by the confusion matrix. Meanwhile, True Positive (TP)、True Negative (TN)、 False Positive (FP)、False Negative (FN) were introduced to describe data. The accuracy rate represents the accuracy of the model’s predictions, and higher its values represent more accurate the model predictions. F1 indicator is obtained through the combination of precision and recall, which represents the balance between precision and recall.

$$Accuracy=\frac{{TP+TN}}{{TP+TN+FP+FN}}$$

$$Precision=\frac{{TP}}{{TP+FP}}$$

$$Recall=\frac{{TP}}{{TP+FN}}$$

$$F1=\frac{{2 \times Precision \times Recall}}{{Precision+Recall}}$$

#### Survival outcome prediction model

**Fig. 3:**
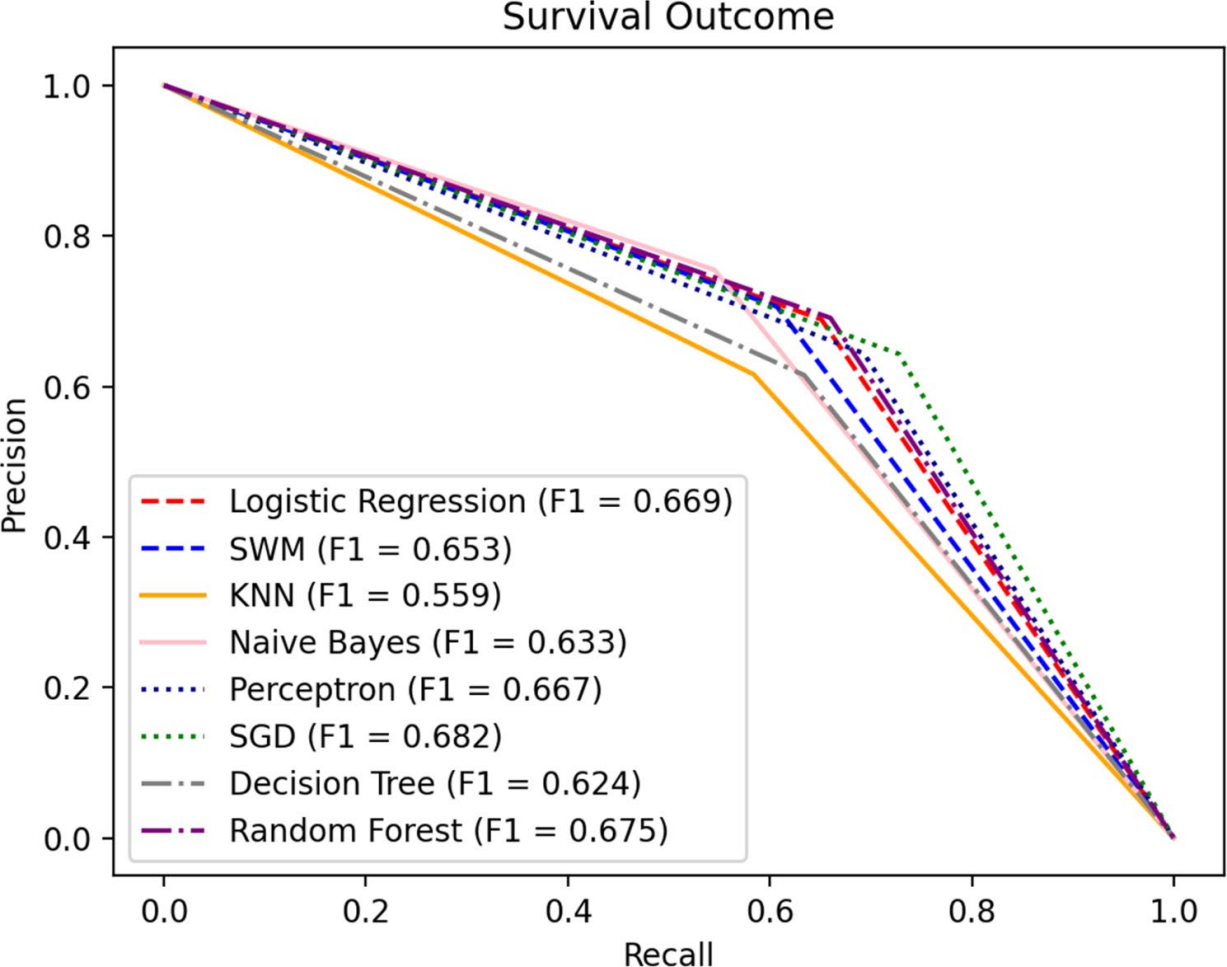
P-R curve of survival outcome

Nine indicators selected above were used as dependent variables of the survival outcome prediction model. In order to obtain a reliable and stable model, we used the 10-fold cross-validations to test the model performances, enabling the models to match the training dataset as closely as possible. The results obtained by the eight machine learning methods were shown in the Table 7. The P-R curves is shown in the Fig. 3.

**Table 7:** prognosis prediction model

		ACC	Precision	Recall	F1	AUC
survival outcome prediction model	Logistic Regression	0.661	0.689	0.651	0.669	0.665
	SVM	0.659	0.706	0.607	0.653	0.665
	KNN	0.596	0.616	0.584	0.559	0.597
	Naive Bayes	**0.669**	0.755	0.545	0.633	**0.679**
	Perceptron	0.596	0.644	0.693	0.667	0.591
	SGD	0.592	0.643	0.728	**0.682**	0.631
	Decision Tree	0.600	0.615	0.634	0.624	0.593
	Random Forest	**0.669**	0.691	0.660	0.675	0.662
30-day renal recovery prediction model	Logistic Regression	0.632	0.446	0.165	0.241	0.534
	SVM	0.63	0.005	0.029	0.037	0.5
	KNN	0.654	0.535	0.534	0.535	0.631
	Naive Bayes	0.569	0.454	0.896	**0.603**	0.632
	Perceptron	0.632	0.293	0.218	0.250	0.524
	SGD	0.637	0.365	0.352	0.358	0.571
	Decision Tree	0.581	0.456	0.451	0.453	0.555
	Random Forest	**0.709**	0.637	0.493	0.556	**0.665**
90-day renal recovery prediction model	Logistic Regression	**0.852**	0.852	1.000	**0.920**	0.500
	SVM	**0.852**	0.852	1.000	**0.920**	0.500
	KNN	**0.852**	0.876	0.959	0.916	**0.621**
	Naive Bayes	0.784	0.876	0.842	0.859	0.604
	Perceptron	0.644	0.867	0.733	0.794	0.517
	SGD	0.818	0.849	0.965	0.903	0.482
	Decision Tree	0.816	0.878	0.901	0.889	0.617
	Random Forest	**0.852**	0.851	0.988	0.914	0.494

Note: Bold indicates better performance

In the prediction model for survival, Naive Bayes and Random Forest have better performance on the accuracy index, with a 1.21–13.00% increase over the rest of the methods. As for the F1 index, SGD has better performance, increasing by 1.04–22.00% compared to the rest of the methods. Considering the AUC index, Naive Bayes performs well, with a 2.11–14.89% increase over the rest of the methods. Overall, Naive Bayes has a good performance in the prediction model for survival.

#### 30-day renal recovery prediction model

**Fig. 4:**
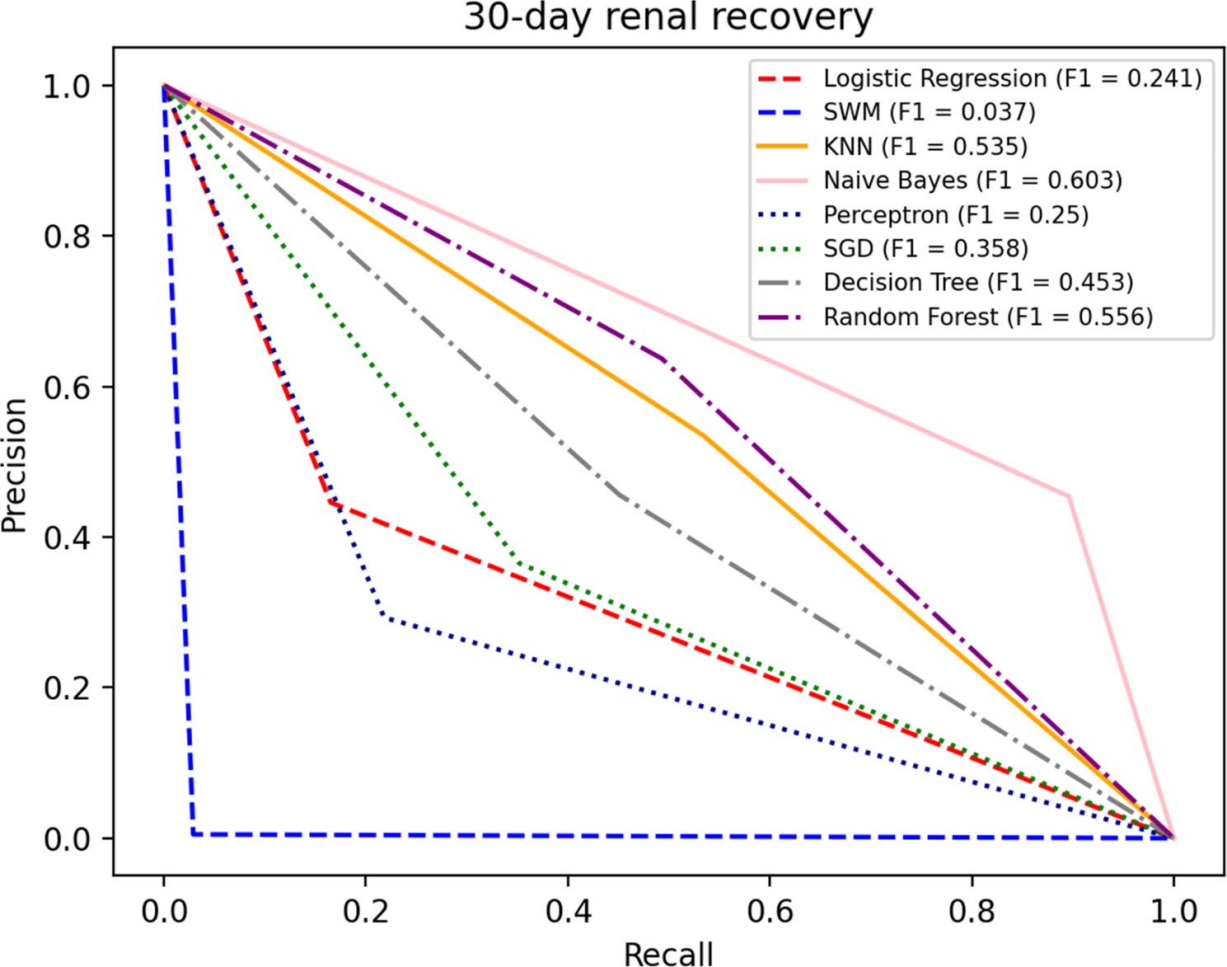
P-R curve of 30-day renal recovery

Eight indicators selected above were used as dependent variables of the 30-day renal recovery prediction model. We used the 10-fold cross-validations to test the model performances. The results obtained by the eight machine learning methods were shown in the Table 7. The P-R curves is shown in the Fig. 4.

In the prediction model for 30-day renal recovery, Random Forest has better performance on the accuracy index, with a 11.30–24.60% increase over the rest of the methods. As for the F1 index, Naive Bayes has better performance, increasing by 8.45–150.21% compared to the rest of the methods, which was obtained after excluding SVM method. SMV method performs poorly on Precision, Recall and F1 indices, this may be due to difference in positive and negative sample sizes, especially when the negative sample size is significantly greater than the positive sample size. Considering the AUC index, Random Forest performs well, with a 5.22–33.00% increase over the rest of the methods. Overall, Random Forest has a good performance in the prediction model for 30-day renal recovery.

#### 90-day renal recovery prediction model

**Fig. 5:**
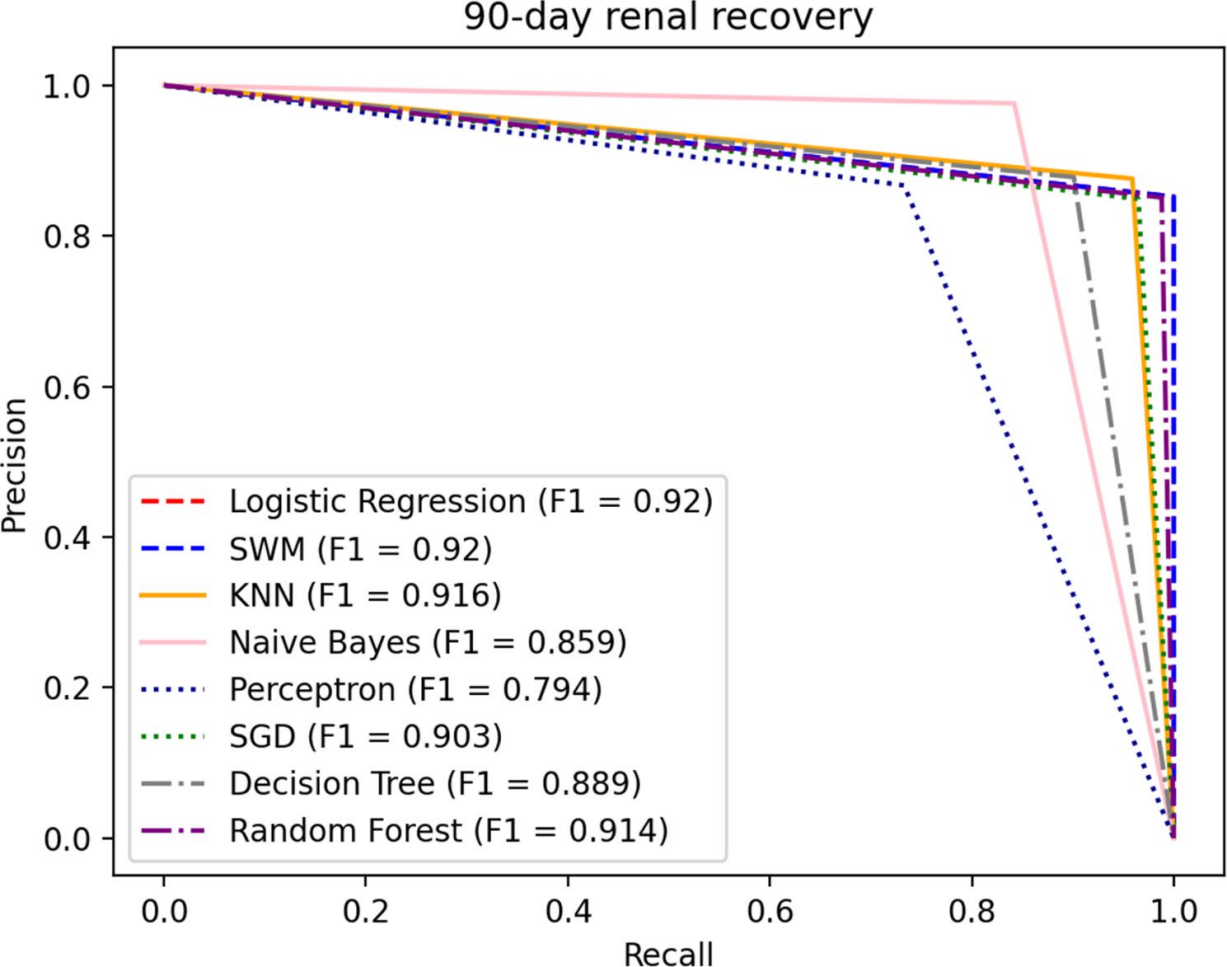
P-R curve of 90-day renal recovery

Hematocrit, Chlorine, Total protein, Uric acid, blood Phosphorus, Prothrombin time, CKD stage, and Diabetes were used as dependent variables of the 90-day renal recovery prediction model. Because of the lesser data, we used the 10-fold cross-validations to test the model performances. The results obtained by the eight machine learning methods were shown in the Table 7. The P-R curves is shown in the Fig. 5.

In the prediction model for 90-day renal recovery, Logistic Regression, SVM, KNN and Random Forest have better performance on the accuracy index, with a 4.16–32.30% increase over the rest of the methods. As for the F1 index, Logistic Regression and SVM have better performance, increasing by 0.44–15.87% compared to the rest of the methods. Considering the AUC index, KNN performs well, with a 0.65–28.84% increase over the rest of the methods. Overall, KNN has a good performance in the prediction model for 90-day renal recovery.

## Discussion

This is the first attempt to use machine learning to establish prognosis prediction models for severe AKI patients who received PIRRT, and Naive Bayes has a good performance in the prediction model for survival, Random Forest has a good performance in 30-day renal recovery prediction model, while for 90-day renal recovery prediction model, it’s KNN.

Specifically, Sodium, Total protein, LDH, Phosphorus, Thrombin time, Liver cirrhosis, CKD stage, number of vital organ injuries, and AKI stage entered the prognostic prediction model of survival outcome. The indices used to predict 30-day renal recovery include Sodium, Total protein, LDH, blood Phosphorus, Thrombin time, Diabetes, PICC, AKI stage. The indices that could predict 90-day renal recovery include Hematocrit, Chlorine, Total protein, Uric acid, blood Phosphorus, Prothrombin time, CKD stage, and Diabetes.

As mentioned above, AKI severity is related to the prognosis, and the same conclusion is reached in this study, meanwhile, attention should be paid to serum electrolytes. Previous studies on serum Sodium were mainly focused on CKD patients since they are prone to abnormal serum Sodium levels due to their weakened ability to maintain water homeostasis, while a sharp decline in renal function can lead to acute changes in serum Sodium levels, which may result in higher mortality and often require immediate treatment to avoid severe neurological complications [13]. Therefore, AKI and abnormal serum Sodium have been under intensive focus recently. In a large retrospective study, a Cox proportional hazards model of 13,621 ICU patients with AKI showed a U-shaped association between Sodium levels and AKI survival [14]. Lee et al. and Edward et al. confirmed similar conclusions [15, 16]. Although Calcium and Phosphorus Metabolism is one of the prognostic factors of CKD, we observed that serum Phosphorus is related to the prognosis of the severe AKI population [17]. Serum phosphorus can be an early biomarker for AKI prediction during pediatric cardiac surgery [18]. Moreover, this study indicates that serum Chlorine is related to the prognosis of AKI patients received PIRRT, which is consistent with the conclusion of Sadan et al. [19].

In the present study, Diabetes is associated with the prognosis of the subjects. Previously, diabetes was associated with CKD, and diabetic nephropathy is the most common cause of end-stage renal disease worldwide. However, some studies have confirmed that diabetic patients are more likely to develop AKI than non-diabetic patients and that diabetes is a major risk factor for AKI [16, 20].

Nevertheless, the present study has several limitations: ① patients may have errors in judging urine volume after discharge. ② Although scientific algorithms have been used for modeling and model verification, this study still has shortcomings in the retrospective design.③ This is a single-center observational study requiring a multicenter external cohort with a large sample size to verify and improve the model. ④ The influence of the specific PIRRT scheme on prognosis was ignored. ⑤ Due to the uneven sample size of positive and negative samples, the accuracy and other indices of the prediction models were influenced.

## Conclusions

In this study, for patients with severe AKI who received PIRRT, the short-term mortality rate was 51.93%, the 30-day renal recovery rate was 30.43%, and the 90-day renal recovery rate was 33.06%. Machine learning can not only screen out factors influencing the short-term prognosis, but also establish prediction models to optimize the risk assessment of these people. These predictors are easy to obtain in the early stage of AKI and attention should be paid to serum electrolytes. Naive Bayes has a good performance in the prediction model for survival, Random Forest has a good performance in 30-day renal recovery prediction model, while for 90-day renal recovery prediction model, it’s K-Nearest Neighbor.

## Electronic supplementary material

Below is the link to the electronic supplementary material.

Supplementary Material 1

## Data Availability

The datasets used and/or analyzed during the current study are available from the corresponding author upon reasonable request.

## Abbreviations

ACC: Accuracy
AKI: Acute kidney injury
AUC: Area Under Curve
CHD: Coronary heart disease
CKD: Chronic kidney disease
COPD: Chronic obstructive pulmonary disease
CRRT: Continuous renal replacement therapy
GFR: Glomerular filtration rate
IHD: Intermittent hemodialysis
KNN: K-Nearest Neighbor
LDH: Lactate dehydrogenase
PICC: Peripherally inserted central catheter
PIRRT: Prolonged intermittent renal replacement therapy
RRT: Renal replacement therapy
Scr: Serum creatinine
SGD: Stochastic Gradient Descent
SVM: Support Vector Machines
