# Supplementary material for: Short-term prognostic models for severe acute kidney injury patients receiving prolonged intermittent renal replacement therapy based on machine learning
Source: BMC Med Inform Decis Mak. 2023 Jul 24;23:133. doi: 10.1186/s12911-023-02231-2 (PMC10367369; doi:10.1186/s12911-023-02231-2)
Supplement: Supplementary file 1 — Supplementary Material 1 [file 12911_2023_2231_MOESM1_ESM.docx]

**Short-term prognostic models for severe acute kidney injury patients receiving prolonged intermittent renal replacement therapy based on machine learning**

**Running title:** Prognostic models of severe AKI patients receiving PIRRT based on machine learning

Wenqian Wei ^1#^, Zhefei Cai ^2#^, Lei Chen ^1#^, Weijie Yuan ^1^, Yingle Fan ^2*^, Shu Rong ^1*^,

^1^Department of Nephrology, Shanghai General Hospital, Shanghai Jiao Tong University School of Medicine, Shanghai, China

^2^Hangzhou Dianzi University, Hangzhou, China

^#^These authors have contributed equally to this work.

***Corresponding author:**

Shu Rong

Department of Nephrology, Shanghai General Hospital, Shanghai Jiao Tong University School of Medicine, Shanghai, China,

E-mail: sophiars@126.com

Tel: +86-13681811669

Yingle Fan

Hangzhou Dianzi University, Hangzhou, China,

E-mail: fan@hdu.edu.cn

**Supplementary Tables**

**Supplementary Table 1 Correlation between data and 30-day renal recovery**

|  | Age | Prothrombin time | Thrombin time | Phosphorus |
| --- | --- | --- | --- | --- |
| 30-day | 0.253 | 0.481 | 0.386 | 0.630 |
|  | Creatinine | GFR | Platelet hematocrit | Hematocrit |
| 30-day | 0.964 | 0.961 | 0.489 | 0.606 |
|  | Hemoglobin | Potassium | Sodium | Chlorine |
| 30-day | 0.615 | 0.532 | 0.457 | 0.520 |
|  | Total protein | Albumin | Globulin | LDH |
| 30-day | 0.746 | 0.638 | 0.652 | 0.814 |
|  | Uric acid | Urea | Calcium | Magnesium |
| 30-day | 0.927 | 0.915 | 0.435 | 0.310 |
|  | Hypertension | Diabetes | Nephropathy history | CKD stage |
| 30-day | 0.014 | 0.043 | <0.001 | <0.001 |
|  | PICC | PIRRT frequency | AKI stage |  |
| 30-day | 0.001 | 0.012 | <0.001 |  |

**Supplementary Table 2 Correlation between data and 90-day renal recovery**

|  | Age | Phosphorus | Prothrombin time | GFR stage |
| --- | --- | --- | --- | --- |
| 90-day | 0.475 | 0.774 | 0.545 | 0.204 |
|  | Potassium | Platelet hematocrit | Hematocrit | Hemoglobin |
| 90-day | 0.872 | 0.596 | 0.864 | 0.849 |
|  | Sodium | Chlorine | Total protein | Albumin |
| 90-day | 0.774 | 0.578 | 0.864 | 0.751 |
|  | Globulin | LDH | Uric acid | Urea |
| 90-day | 0.774 | 0.879 | 0.879 | 0.955 |
|  | Calcium | Magnesium | Thrombin time |  |
| 90-day | 0.540 | 0.568 | 0.607 |  |
|  | Diabetes | CKD stage | Diuretic | ARB |
| 90-day | 0.001 | <0.001 | <0.001 | 0.004 |
|  | β-blockers |  |  |  |
| 90-day | 0.004 |  |  |  |
